# Molecular prevalence of equine parvovirus hepatitis in healthy horses from the Northern region of the state of Rio Grande do Sul, Brazil

**DOI:** 10.1007/s11259-026-11251-y

**Published:** 2026-05-09

**Authors:** Taline Scalco Picetti, Andreza Soriano Figueiredo, Júlia Trevizan Segalin, Nicole Lopes Mostardeiro, Gabriele de Almeida, Rafael Frandoloso, Luiz Carlos Kreutz

**Affiliations:** 1 Laboratory of Advanced Microbiology and Immunology, School of Agricultural Sciences, Innovation and Business, University of Passo Fundo, Campus I, Bairro São José, Passo Fundo, 99052-900 RS Brazil; 2 Laboratory of Technological Development in Virology, Oswaldo Cruz Institute, Fiocruz, Rio de Janeiro, Brazil

**Keywords:** Emerging viruses, Hepatitis, Horse, Diagnosis, Epidemiology, PCR

## Abstract

The equine parvovirus hepatitis (EqPV-H) was initially linked to fatal cases of equine serum hepatitis but, more recently, it has also been detected in sick or asymptomatic horses around the world, including in a limited number of horses in southeastern Brazil. In this study we aimed to assess the prevalence of EqPV-H DNA in the serum of healthy horses in the northern region of Rio Grande do Sul state, Brazil. For this purpose, we used 1000 serum samples collected from horses from 40 municipalities and organized them into 200 pools of five samples each, based on origin and age. Using a nested polymerase chain reaction (nPCR), we detected the EqPV-H DNA in 69 of the pools representing 34.5% of the total, with positive samples found in 26 (65%) of the municipalities. The prevalence did not differ significantly by age group (*p* = 0.52). The nucleotide and phylogenetic analysis conducted with the partial NS1 sequence obtained from five serum pools revealed a high degree of genetic conservation with previous worldwide isolates, indicating specific nucleotide variability present in strains from Brazil and Argentina but absent in strains from other countries. Our study provides the first evidence that EqPV-H is widespread in horses in the region and it should be considered in suggestive clinical cases of hepatitis.

**Supplementary Information:**

The online version contains supplementary material available at 10.1007/s11259-026-11251-y.

## Introduction

Equine serum hepatitis, also known as Theiler’s disease, was first described in South Africa in 1918 (Theiler 1918) and subsequently reported in horses worldwide, usually following the administration of horse-derived biological products (Ramsauer et al. 2021). Recently, the etiology of equine serum hepatitis was linked to a novel non-enveloped single-stranded DNA virus classified within the *Parvoviridae* family (genus *Copiparvovirus*, *species Copiparvovirus ungulate 6*) and named equine parvovirus hepatitis (EqPV-H) (Divers et al. 2018). EqPV-H is a non-enveloped single-stranded DNA virus with a genome comprising 5,308 nucleotides (nt), displaying two open reading frames (ORFs); ORF1 encodes a non-structural protein (NS), and ORF2 encodes the structural proteins of the virus (Divers et al. 2018).

Following infection and an incubation period of 4 to 12 weeks, EqPV-H can be found in the blood and several organs such as liver, lung, spleen, lymph nodes, bone marrow, kidney and heart during the acute phase of hepatitis (Tomlinson et al. 2020). Yet, in some horses, EqPV-H can be detected in the blood of asymptomatic horses for up to five years (Reinecke et al. 2021). The primary clinical signs observed during the acute phase of hepatitis are jaundice, lethargy, loss of appetite, and neurological clinical signs such as blindness, obtundation, ataxia, and pressing the head against objects (Tomlinson et al. 2019a, b; Baird et al. 2020). The clinical form can progress to acute hepatic necrosis with elevated mortality (Divers et al. 2018; Tomlinson et al. 2020; Vengust et al. 2020a) but in most cases, the infection is self-limiting, and affected horses may show minimal or even or no increase in liver enzyme activity (Tomlinson et al. 2020). EqPV-H spreads intermittently via the oral, nasal, fecal, and semen routes (Yoon et al. 2022a). On farms with infected mares, most foals become infected in their first year of life, possibly through contact with the infected mares or because of treatment with hyperimmune plasma to prevent infection by *Rhodococcus equi* (Tomlinson et al. 2020). Foals exhibit greater resistance to infection and generally do not develop disease in its severe form; in contrast, the prevalence of infection is usually higher in older animals (Badenhorst et al. 2022). The possibility of EqPV-H infecting other equids has been examined, and antibodies against the virus were detected only in donkeys. To date, there is no evidence of infection in humans (Reinecke et al. 2021).

In several studies, EqPV-H was detected in asymptomatic horses which, in this case, might act as silent carriers, facilitating the spread of the virus and contributing to the risk of, and outbreaks of, fatal Theiler’s disease. While most infected horses show no signs, the virus might be found in 7 to 17% of healthy horse populations worldwide. Up to now, EqPV-H has been detected in the United States (Divers et al. 2018), Canada (Baird et al. 2020; Papapetrou et al. 2023), China (Lu et al. 2018; Xie et al. 2020), Germany (Meister et al. 2019b), Slovenia (Vengust et al. 2020b), France and Australia (Fortier et al. 2024), Austria (Badenhorst et al. 2022), South Korea (Lee et al. 2021; Yoon et al. 2022b) and Argentina (Olguin-Perglione et al. 2024). In Brazil, a single epidemiological study analyzed 96 equine serum samples taken from 15 farms in various municipalities from Rio de Janeiro state (southeast of Brazil) between 2013 and 2016, and the EqPV-H DNA was detected in 12 (12.5%) serum samples from 7 (46.6%) farms (de Moraes et al. 2022). EqPV-H was also detected in batches of equine serum from Germany, Canada, USA, Italy, New Zealand (Meister et al. 2019b), Argentina (Olguin-Perglione et al. 2024) and Brazil (Paim et al. 2019).

No other studies have been conducted in Brazil concerning the epidemiology of EqPV-H. The lack of epidemiological data on EqPV-H prevents the assessment of its impact on liver disease and mortality in horses. Thus, the objective of this study was to assess the presence and distribution of EqPV-H in healthy horses in the northern region of the state of Rio Grande do Sul.

## Materials and methods

### Serum samples

Serum samples were collected from healthy horses over 6 months old by field veterinarians between August and December 2022 and sent to the Veterinary Diagnostic Laboratory at the University of Passo Fundo for the diagnosis of Equine Infectious Anemia and Equine Glanders. Following the completion of the requested tests and obligatory storage period, we randomly selected 1000 serum samples from 40 municipalities located in the northern region of the state of Rio Grande do Sul state (RS), Brazil. Information on horse geographical origin, sex, age and race were taken from the horse documents sent along with the sample for the obligatory exams. The number of samples needed for the study was calculated using Epi Info software and the total number of horses (12,678 horses) in the municipalities; the prevalence of EqPV-H was estimated at 10% based on the average prevalence of EqPV-H genome detection in the serum of healthy horses, as reported in previous studies (Divers et al. 2018; Meister et al. 2019b; Xie et al. 2020; Badenhorst et al. 2022). Considering a 99% confidence level and an acceptable margin of error of 1%, it would be necessary to evaluate at least 234 samples. Therefore, the sample size used in this study exceeds the minimal requisite for epidemiological surveillance investigation. The number of horses registered in the municipalities (IBGE 2023) and the number of samples selected for the study per municipality are displayed in Table S1.

### Organization of samples and DNA extraction

Serum aliquots (100 µL) from five horses from the same municipality and age category (under 6 years; between 6 and 10 years, and above 11 years) were grouped into a single sample (pool), totaling 200 pools that were used for DNA extraction using a commercial kit (Whole Blood Genomic DNA purification, Ludwig Biotecnologia). DNA was eluted in 30 µL of ultrapure water and stored (-20 °C) until use.

### Detection of EqPV-H DNA

The EqPV-H DNA was detected by nested polymerase chain reaction (nPCR), using primers targeting the gene encoding the EqPV-H NS1 protein, and cycling conditions as described previously (Divers et al. 2018) and confirmed for EqPV-H detection in Brazil (de Moraes et al. 2022). Negative controls (ultrapure water) and DNA controls known to be positive for the presence of EqPV-H DNA (de Moraes et al. 2022) were used in all amplifications. The nPCR product was analyzed by agarose gel electrophoresis (1.5%). Samples that contained a DNA fragment with 435 base pairs were considered positive.

### DNA sequencing and phylogenetic analysis

The nPCR product (435 bp) from selected samples was separated by low-melting agarose gel electrophoresis, purified using a commercial kit (EasyPure Quick Gel Extraction, TransGen Biotech, China), and sequenced in both directions. The sequences of nucleotides obtained were submitted to the GenBank database under the accession numbers PX452558 to PX452562. Published sequences for comparative analysis were retrieved using the online server provided by the NCBI GenBank database (https://www.ncbi.nlm.nih.gov/nucleotide/), accessed on 29 August 2025. Sequences were aligned with ClustalW and analyzed in MEGA 10 (Kumar et al. 2018) and the web-based application Weblogo (https://weblogo.berkeley.edu/logo.cgi) (Crooks et al. 2004), accessed on 10 October 2025. The appropriate substitution model was indicated using the best DNA/protein model finding tool of MEGA 10. The Maximum Likelihood method was performed with 1000 bootstraps using the Kimura 2-parameter model (K2 + G) (Kimura 1980). Nucleotide identities were analyzed using BLAST (https://blast.ncbi.nlm.nih.gov/blast/Blast. cgi), accessed on 05 September 2025.

## Results

**Fig. 1:**
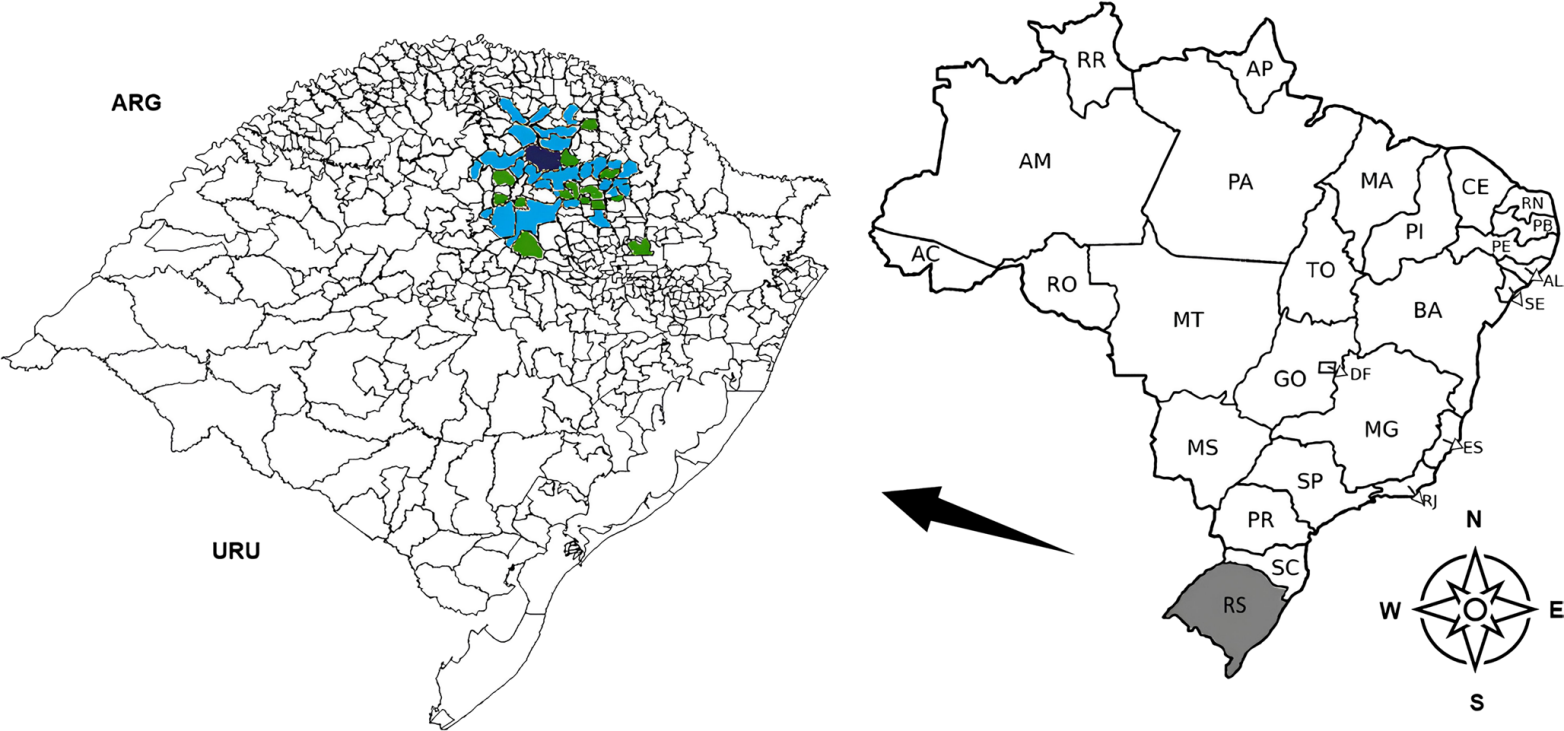
Geographic distribution of the serum samples used to estimate the molecular prevalence of Equine Parvovirus Hepatitis in horses. EqPV-H was detected in 26 municipalities (blue painted); dark blue indicates the location of the University of Passo Fundo. Municipalities with no horses positive to EqPV-H are indicated with green color. ARG, Argentina, URU, Uruguay

Our study analyzed 1000 serum samples from healthy horses distributed across 40 municipalities in the northern part of RS (Fig. 1) together housing 12,678 registered horses (IBGE, 2023). The samples were organized into 200 pools (5 samples/pool) which were subsequently analyzed by nPCR for the presence of EqPV-H DNA. Of these, 69 pools (34.5%) across 26 (65%) municipalities yielded a DNA fragment containing approximately 435 base pairs and were considered positive for EqPV-H.

The geographical distribution of the horse population, pooling of the serum samples, positive pools and positive municipalities for EqPV-H are provided in the Supplementary Material (Table S1). Concerning the horse gender, 568 (56.8%) of the samples were obtained from females and 432 (43.2%) from males. Whitin younger horses (> 6 months < 6years) 40% of the serum pools tested positive to EqPV-H while a lower prevalence of positive serum pools was found within horses aged 6 to 10 years (31.8%) and those over 11 years (31.9%) (Table 1).

**Table 1:** Distribution of serum samples and serum pooled samples positive for the presence of EqPV-H DNA according to horse age range

Age range	Number of serum samples	Number of pools	Number of positive pools	Percentage of positive pools
> 6 months ≤ 6 years	325	65	26	40.0
6 to 11 years	440	88	28	31.8
≥ 11 years	235	47	15	31.9
**Total**	**1000**	**200**	**69**	**34.5**

The sequencing of the DNA fragment and inspection of the obtained chromatograms (391 bp) indicated the presence of eight positions with overlapping nucleotide peaks, identified as Y (C or T) and R (A or G), suggesting the amplification of different variants present in the pooled serum samples. The positions, according to provisional REFSEQ NC_076001, and further comprehensive information are provided in Table S2 (Supplementary Material). The evaluation of nucleotide variability of 182 EqPV-H sequences retrieved from the GenBank database showed that three mixed-bases are present in isolates from Brazil and Argentina but absent in isolates from Australia, Austria, Canada, China, France, South Korea, and the United States (Figure S1 of Supplementary Material).

**Fig. 2:**
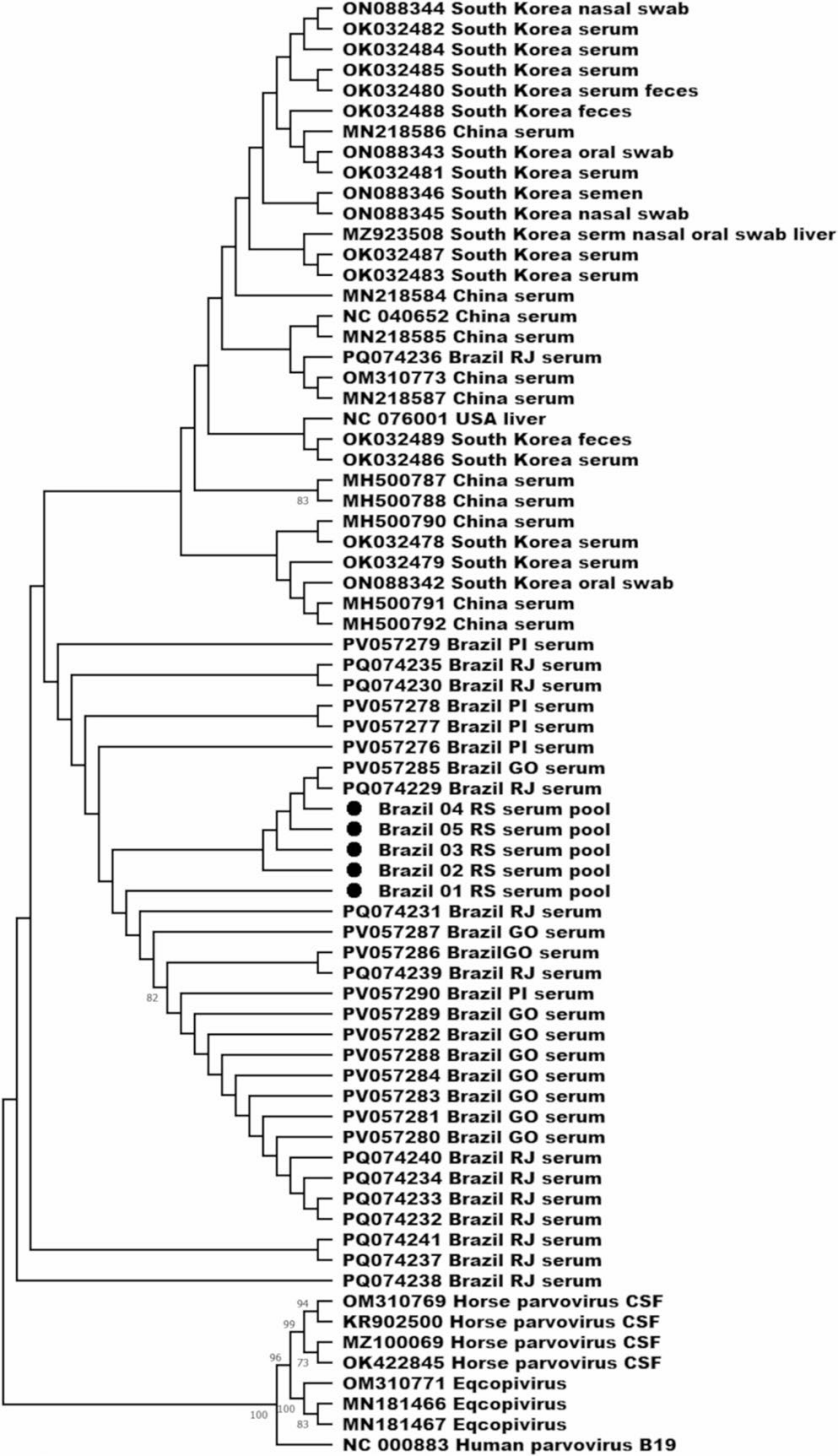
Phylogenetic tree (cladogram) based on the analysis of the partial (391) NS1 gene of the *Copiparvovirus* genus of the *Parvoviridae* family, including EqPV-H isolates of this study (•), the provisional REFSEQ NC_076001 and NC_040652, EqPV-H sequences available in the GenBank database, sequences of other viruses affecting equids, the eqcopivirus (*Copiparvovirus ungulate7*) and horse parvovirus CSF (*Copiparvovirus ungulate 8*), and the reference sequence of human parvovirus B19 (*Erythroparvovirus* genus) as outgroup. Sequences are described with accession number, country, state (Brazilian sequences), and biological sample. Tree was built using the Maximum Likelihood method and Kimura 2-parameter model (K2 + G) with 1,000 replicates

A phylogenetic tree was built using EqPV-H and other members of the *Copiparvovirus* genus that infect equid species, including Eqcopivirus (MN181466, MN181467, OM310771) and horse parvovirus CSF (KR902500, MZ100069, OK422845, OM310769). Human parvovirus B19 (NC_000883) was included as an outgroup (Fig. 2).

## Discussion

Here we demonstrate that EqPV-H is widely distributed in healthy horses in the north region of Rio Grande do Sul state, Brazil. Out of 40 municipalities included in the study, 26 (65%) harbored horses that were positive to EqPV-H. The use of pooled samples allowed us to expand the range of the study but diminished the capacity to accurately estimate the prevalence of positive animals. Nonetheless, at the individual level, the prevalence of horses containing the EqPV-H genome was estimated based on the number of serum pools. Out of 200 pools, we found 69 (34,5%) with detectable EqPV-H DNA; considering that each of the 69 positive pools contained at least one positive sample, the minimum prevalence of horses harboring the EqPV-H genome was estimated to be 6.9% (69 sample/1000). Conversely, some of the polls deemed negative could have traces of DNA undetected by the nPCR assay. In addition, some positive pools could have more than one positive serum sample. Thus, in both situations, the prevalence of EqPV-H in these equine population could exceed 6.9%. We considered testing all five samples from each of the 69 positive pools (*n* = 345 samples) but determined that it would not be cost-effective. In a hypothetical scenario where all five samples from the 69 pools could be positive, the maximum prevalence would be 34.5%, much exceeding the global reports for asymptomatic horses. The northern half of Rio Grande do Sul comprises around 330 to 350 municipalities exhibiting similar geographical and agricultural traits, including equine density (IBGE, 2023); hence, the incidence of EqPV-H throughout the region is expected to align with the findings presented herein.

In a previous investigation in Brazil, with a limited number of samples, the EqPV-H genome was detected in 12 (12.5%) of 96 serum samples (de Moraes et al. 2022). Nonetheless, the minimum prevalence of EqPV-H infection estimated in our study (6.9%) aligns closely with findings from other studies conducted worldwide, such as those in Germany (7.14%Meister et al. 2019a) and Austria (8.9%) (Badenhorst et al. 2022), but slightly lower than the prevalence found in horses in China (11.9%) (Xie et al. 2020), USA (13.0%)(Divers et al. 2018) and Canada (15.9%) (Papapetrou et al. 2023). Conversely, in farms with a history of horses with liver illness, the prevalence of infection was higher; in Canada, for instance, EqPV-H was detected in 47.1% of healthy horses on a farm with a record of acute and fulminant liver disease (Baird et al. 2020). Asymptomatic carriers of EqPV-H act as reservoirs and likely are the source of infection to healthy horses; these carriers shed the virus via nasal, oral and fecal routes posing significant risk as sources of contaminated blood-derived biological products. Indeed, up to 61.1% of commercial serum pools have tested positive to EqPV-H (Meister et al. 2019b; Paim et al. 2019; Olguin-Perglione et al. 2024). Although many infected horses exhibit subclinical hepatitis with mild liver enzyme elevation, EqPV-H has been linked to decreased performance in racehorses. Thus, because undetected virus circulation is heightened by the often-asymptomatic nature of the infection, particularly in older horses, preventive measures suggest the routine use of PCR to detect EqPV-H in horses used to produce biological products, semen collection and reproduction, and during quarantine.

The prevalence of the EqPV-H was reported to be higher in older horses (Badenhorst et al. 2022). Here, we found that there was no correlation (*p* = 0.54) between the horse age range and the presence of EqPV-H DNA in serum samples. Thus, considering that each pool had a single positive sample, as discussed above, the prevalence of EqPV-H infection was 8% in horses under 6 years, 6.4% in those aged 6 to 10 years, and 6.4% in horses over 11 years. Our study analyzed a greater quantity of samples compared to that reported in previous studies (Badenhorst et al. 2022) and encompassed several types of farms and horse breeds, which may have contributed to the similar prevalence observed among the different age groups. Conversely, it is possible that if we had increased the number of age groups while decreasing the interval between them, the prevalence in each age group could have had a different outcome.

To determine the phylogenetic relationships of the partial NS1 sequence obtained in this study (Brazil RS 01 - Brazil RS 05) alongside other available sequences of the equivalent length, we performed nucleotide and phylogenetic analyses involving 28 sequences from Brazil (PQ074229-PQ074241, PV057276-PV057276), 18 sequences from South Korea (MZ923508, OK032479-OK032489, ON088342-ON088346), 11 sequences from China (MH500787, MH500788, MH500790-MH500792, MN218584-MN218587, OM310773, and provisional reference sequence NC_040652), and one sequence from the United States (provisional reference sequence NC_076001). Nucleotide identities exhibited considerable similarity within sequences of this study, ranging from 98.47% to 99.74%, and with Brazilian and other countries, from 95.65% to 99.49% and 96.42% to 99.35%, respectively. The isolates of this study grouped with most of the Brazilian isolates, forming a separate cluster from the European, Asian, and North American isolates. The analysis of nucleotide variability of 182 EqPV-H sequences retrieved from the GenBank database showed that three mixed-bases are present in isolates from Brazil and Argentina but absent in isolates from Australia, Austria, Canada, China, France, South Korea, and the United States (Figure S1 of Supplementary Material). These findings may indicate the possible circulation of slightly divergent strains between Brazil and Argentina, indicative of geographical and/or international linkages, as documented for Australian(Fortier et al. 2024) and Korean strains (Yoon et al. 2021). To confirm this hypothesis, the entire genome of EqPV-H should be analyzed, ideally including data from more South American countries. Overall, the molecular analysis performed in this study confirms the high degree of genetic conservation among the EqPV-H described worldwide(Lu et al. 2018; Meister et al. 2019b; Baird et al. 2020; Reinecke et al. 2021; Badenhorst et al. 2022; de Moraes et al. 2022; Yoon et al. 2022b; Olguin-Perglione et al. 2024) while also highlighted the need for additional research across diverse geographical region to enhance the understanding of the virus distribution and genetic variability.

We acknowledge that a limitation of our study may be the use of DNA extracted from pooled serum sample for sequencing analysis. Even considering that the members of the *Parvoviridae* family present a low rate of mutation, and the cost-benefit of using these samples, the accuracy of detecting nucleotide variability could be reduced. Indeed, as demonstrated in Figure S1, we notice nucleotide ambiguity in several positions within different isolates.

## Conclusion

The results of our study demonstrate, for the first time, the occurrence of EqPV-H in horses in northern region of Rio Grande do Sul state, Brazil. EqPV-H is widely distributed among horses across different municipalities in the region, exhibiting a minimum prevalence of 6.9%. The EqPV-H isolates presented a high degree of genetic conservation with previous Brazilian and other countries’ isolates. This epidemiological study may contribute to identifying liver diseases in horses and the development of new strategies for the prevention and control of this novel viral agent in horses.

## Supplementary Information

Below is the link to the electronic supplementary material.

Supplementary Material 1

Supplementary Material 2

Supplementary Material 3

## Data Availability

No datasets were generated or analysed during the current study.
