# Supplementary material for: Molecular prevalence of equine parvovirus hepatitis in healthy horses from the Northern region of the state of Rio Grande do Sul, Brazil
Source: Vet Res Commun. 2026 May 9;50(4):311. doi: 10.1007/s11259-026-11251-y (PMC13157447; doi:10.1007/s11259-026-11251-y)
Supplement: Supplementary file 1 — Supplementary Material 1 [file 11259_2026_11251_MOESM1_ESM.docx]

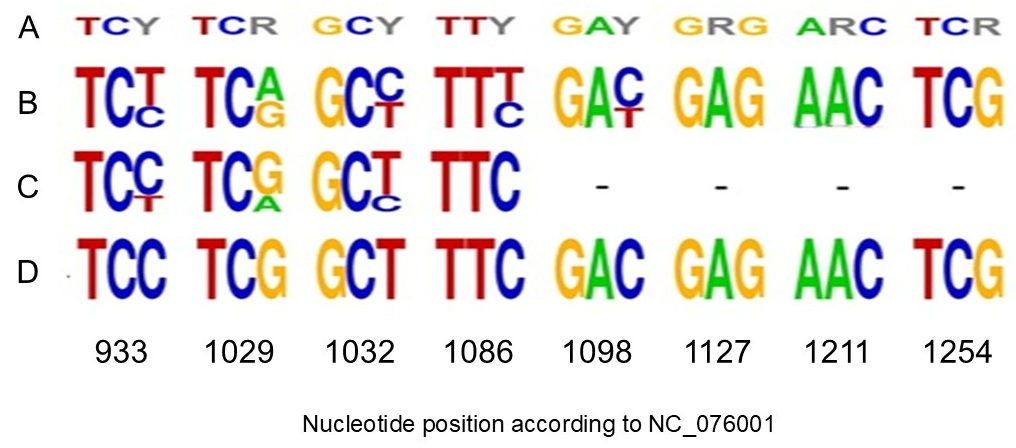


Figure S1. Nucleotide variability present in the positions with mixed-bases described in the EqPV-H sequences of this study, Brazil 01 RS to Brazil 05 RS, along with the variability of EqPV-H sequences available in GenBank database from several countries. Ambiguity in nucleotides 1127, 1211 and 1254 are only found in the Brazilian isolates. **A**) Nucleotide ambiguity described in the isolates Brazil 01 RS to Brazil 05 RS. **B**) Nucleotide variability among 39 isolates from Brazil (MT740217-MT740223, MZ3122307-MZ312310, PQ074229-PQ074241, PV057276-PV057276. **C**) Nucleotide variability among 11 isolates from Argentina (PP408676-PP408687. **D**) Nucleotide variability among 132 isolates from Australia (PP54305-PP44308), Austria (MT559077-MT559081), Canada (MK792429-MK792437, MN397829), China (MH500787-MH500792, MN218583-MN218587, M310773), France (PP544296-PP544304), Germany (MN184860-MN184879, MW828692-MW8287280), South Korea (MZ9235508, OK336512, OK358687-OK358700, OK032478-OK032489, ON088342-ON088346), and United States (NC_076001). The compilation of variability of the sequences was built using the web-based application Weblogo (<https://weblogo.berkeley.edu/logo.cgi>), accessed on 10 October 2025.

The compilation of variability of the sequences was built using the web-based application Weblogo (<https://weblogo.berkeley.edu/logo.cgi>), accessed on 10 October, 2025.

1. Nucleotide ambiguity described in the isolates Brazil 01 RS to Brazil 05 RS.
2. Nucleotide variability among 39 isolates from Brazil (MT740217-MT740223, MZ3122307-MZ312310, PQ074229-PQ074241, PV057276-PV057276
3. Nucleotide variability among 11 isolates from Argentina (PP408676-PP408687.
4. Nucleotide variability among 132 isolates from Australia (PP54305-PP44308), Austria (MT559077-MT559081), Canada (MK792429-MK792437, MN397829), China (MH500787-MH500792, MN218583-MN218587, M310773), France (PP544296-PP544304), Germany (MN184860-MN184879, MW828692-MW8287280), South Korea (MZ9235508, OK336512, OK358687-OK358700, OK032478-OK032489, ON088342-ON088346), and United States (NC_076001)
