# Supplementary material for: Molecular prevalence of equine parvovirus hepatitis in healthy horses from the Northern region of the state of Rio Grande do Sul, Brazil
Source: Vet Res Commun. 2026 May 9;50(4):311. doi: 10.1007/s11259-026-11251-y (PMC13157447; doi:10.1007/s11259-026-11251-y)
Supplement: Supplementary file 2 — Supplementary Material 2 [file 11259_2026_11251_MOESM2_ESM.docx]

Table S1 Detailed information concerning the geographical distribution of the horse population, pooling of the serum samples, positive pools and positive municipalities for EqPV-H in the northern region of Rio Grande do Sul, Brazil.

| **Municipality** | | **horse population** | **serum samples analyzed** | **pooled samples analyzed** |
| --- | --- | --- | --- | --- |
| 1 | Alto Alegre | 48 | 5 | 1 |
| 2 | Barros Cassal | 800 | 10 | 2 |
| 3 | Bento Gonçalves | 369 | 5 | 1 |
| 4 | Camargo | 173 | 5 | 1 |
| 5 | Carazinho | 1.219 | 90 | 18 |
| 6 | Casca | 198 | 5 | 1 |
| 7 | Charrua | 79 | 5 | 1 |
| 8 | Ciríaco | 323 | 55 | 11 |
| 9 | Coxilha | 404 | 30 | 6 |
| 10 | David Canabarro | 223 | 10 | 2 |
| 11 | Ernestina | 263 | 65 | 13 |
| 12 | Espumoso | 636 | 55 | 11 |
| 13 | Gentil | 154 | 15 | 3 |
| 14 | Getúlio Vargas | 150 | 5 | 1 |
| 15 | Guabiju | 148 | 30 | 6 |
| 16 | Guaporé | 265 | 25 | 5 |
| 17 | Ibiraiaras | 233 | 25 | 5 |
| 18 | Marau | 567 | 75 | 15 |
| 19 | Mato Castelhano | 233 | 25 | 5 |
| 20 | Montauri | 46 | 10 | 2 |
| 21 | Mormaço | 75 | 5 | 1 |
| 22 | Muliterno | 51 | 10 | 2 |
| 23 | Não-me-toque | 214 | 35 | 7 |
| 24 | Nicolau Vergueiro | 128 | 15 | 3 |
| 25 | Nova Alvorada | 127 | 25 | 5 |
| 26 | Nova Araçá | 71 | 5 | 1 |
| 27 | Parai | 122 | 25 | 5 |
| 28 | Passo Fundo | 1.784 | 100 | 20 |
| 29 | Pontão | 207 | 15 | 3 |
| 30 | Quatro Irmãos | 159 | 5 | 1 |
| 31 | Ronda Alta | 130 | 30 | 6 |
| 32 | Saldanha Marinho | 103 | 5 | 1 |
| 33 | Santo Antônio do Planalto | 149 | 10 | 2 |
| 34 | São Domingos do Sul | 96 | 10 | 2 |
| 35 | São Jorge | 131 | 40 | 8 |
| 36 | Serafina Corrêa | 186 | 5 | 1 |
| 37 | Sertão | 220 | 10 | 2 |
| 38 | Soledade | 1.941 | 70 | 14 |
| 39 | Tapera | 128 | 5 | 1 |
| 40 | Vila Maria | 125 | 25 | 5 |
|  | **TOTAL** | **12.678** | **1000** | **200** |
