# Supplementary material for: Molecular prevalence of equine parvovirus hepatitis in healthy horses from the Northern region of the state of Rio Grande do Sul, Brazil
Source: Vet Res Commun. 2026 May 9;50(4):311. doi: 10.1007/s11259-026-11251-y (PMC13157447; doi:10.1007/s11259-026-11251-y)
Supplement: Supplementary file 3 — Supplementary Material 3 [file 11259_2026_11251_MOESM3_ESM.docx]

Table S2 Information of the mixed-bases present in five sequences of the EqPV-H isolates.

| **Sequence ID** | **Degenerate nucleotide** | **Location (NC_076001)** | **GenBank acc. number** |
| --- | --- | --- | --- |
| UPF_LDV_19 | Y | 933 | PX452558 |
|  | R | 1029 |  |
|  | Y | 1032 |  |
|  | Y | 1086 |  |
|  | Y | 1098 |  |
|  | R | 1254 |  |
| UPF_LDV_20 | R | 1254 | PX452559 |
| UPF_LDV_21 | Y | 933 | PX452560 |
|  | Y | 1032 |  |
|  | Y | 1086 |  |
|  | Y | 1098 |  |
|  | R | 1211 |  |
|  | R | 1254 |  |
| UPF_LDV_22 | R | 1254 | PX452561 |
| UPF_LDV_23 | R | 1254 | PX452562 |
